# Unanticipated Testicular Involvement in Prostate Carcinoma: A Case Report

**DOI:** 10.7759/cureus.104118

**Published:** 2026-02-23

**Authors:** Simha Swaraj Sirivela, Vivek Patil, Prashanth Reddy Yelsani, Mounish Nuthalapati, Adithya V Reddy, Midhun Mohan

**Affiliations:** 1 Urology, Amrita Institute of Medical Sciences, Kochi, IND

**Keywords:** androgen-deprivation therapy, lower urinary tract symptoms, metastasis, metastatic prostate carcinoma, orchidectomy, pet-ct scan, prostate adenocarcinoma (pca), prostate-specific antigen (psa), testicular metastasis, transrectal ultrasound (trus)

## Abstract

Prostate carcinoma commonly metastasizes to distant organs; however, testicular involvement is exceedingly rare and may be overlooked in routine clinical practice. We report the case of a 78-year-old man with high-grade prostate adenocarcinoma who presented with a three-month history of painless hematuria and lower urinary tract symptoms (LUTS). Initial evaluation revealed a prostate-specific antigen (PSA) level of 11.01 ng/mL, and multiparametric MRI demonstrated locally advanced disease. A transrectal ultrasound (TRUS)-guided biopsy confirmed adenocarcinoma with a Gleason score of 10. Whole-body gallium-68 (⁶⁸Ga) prostate-specific membrane antigen positron emission tomography-computed tomography (PSMA PET-CT) identified a PSMA-avid prostatic lesion with extension into the seminal vesicles and bladder, along with two focal PSMA-avid deposits in the left testis. Bilateral orchidectomy was performed, and histopathological evaluation confirmed metastatic prostate adenocarcinoma within the left testis. The confirmation of distant metastatic involvement prompted the intensification of systemic therapy with abiraterone and prednisolone, resulting in a decline in PSA to 4.63 ng/mL at four-month follow-up. This case highlights the importance of comprehensive staging in high-grade prostate cancer and demonstrates the clinical utility of PSMA PET-CT in detecting atypical metastatic sites such as the testes, thereby guiding appropriate surgical and systemic treatment decisions.

## Introduction

Testicular metastases from solid tumors are exceedingly rare. Early autopsy evaluations reported an incidence ranging from 0.02% to 2.5%, underscoring their rarity across malignancies [1]. Although prostate carcinoma is the most frequent primary tumor associated with secondary testicular involvement, true metastatic spread to the testis remains uncommon. A large autopsy series of 1,589 men with prostate cancer demonstrated testicular metastases in only approximately 0.5% of cases, even among those with advanced disease burden [2]. Most documented cases occur incidentally and are identified through orchiectomy or postmortem examination rather than clinical presentation [3]. As a result, clinically evident testicular metastases from prostate adenocarcinoma are reported mainly as isolated case reports or small series [4-6].

Several mechanisms have been proposed to explain this rare metastatic pattern, including retrograde venous dissemination, lymphatic spread, arterial embolization, and intraductal migration through the vas deferens [7,8]. With the advent of prostate-specific membrane antigen (PSMA) positron emission tomography-computed tomography (PET-CT), the detection of atypical sites of metastasis has significantly improved. Evidence from prospective and observational studies demonstrates that PSMA PET-CT provides superior diagnostic accuracy compared to conventional imaging for identifying metastatic deposits, including unusual or small-volume lesions [9-11].

We report a rare case of testicular metastasis from prostate adenocarcinoma detected on PSMA PET-CT and confirmed histologically. This case highlights the importance of considering metastatic disease in elderly men presenting with testicular lesions, especially in the context of advanced or high-grade prostate cancer.

## Case presentation

A 78-year-old male patient presented with a three-month history of intermittent hematuria and lower urinary tract symptoms (LUTS). He had initially sought care at another hospital, where he was started on medical therapy with silodosin + dutasteride for symptomatic relief. Digital rectal examination at that time revealed a grade 2, benign-feeling prostate. His prostate-specific antigen (PSA) at presentation was 11.01 ng/mL (reference range: 0.0-4.4).

The multiparametric MRI of the pelvis demonstrated a grossly enlarged prostate gland measuring 76 × 73 × 67 mm. The left half of the gland contained a large, irregular mass measuring 7 × 6 × 5 cm, showing diffusion restriction and heterogeneous differential enhancement. The lesion extended across the midline into the right half of the gland and exhibited the invasion of the left posteroinferior bladder wall and the left seminal vesicle, with no definite rectal invasion. No significant pelvic lymphadenopathy was noted. Magnetic resonance spectroscopy demonstrated increased choline peaks with reduced citrate, producing altered metabolic ratios suggestive of malignancy. Based on these imaging findings, the clinical stage at presentation was assigned as cT4N0.

**Figure 1:**
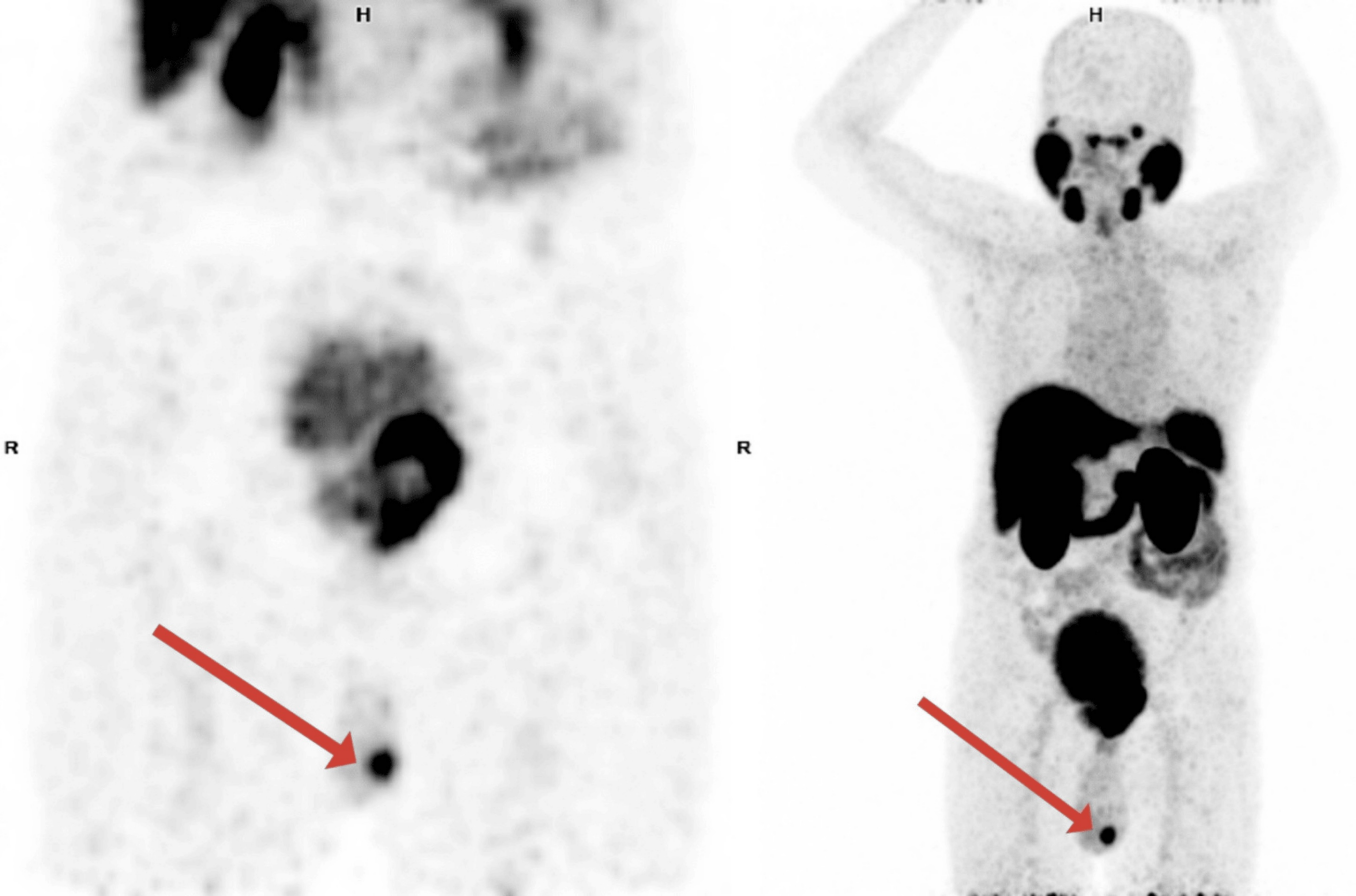
Coronal PET-only slice showing PSMA-avid enhancing focal lesions involving the left testis. PET, positron emission tomography; PSMA, prostate-specific membrane antigen

**Figure 2:**
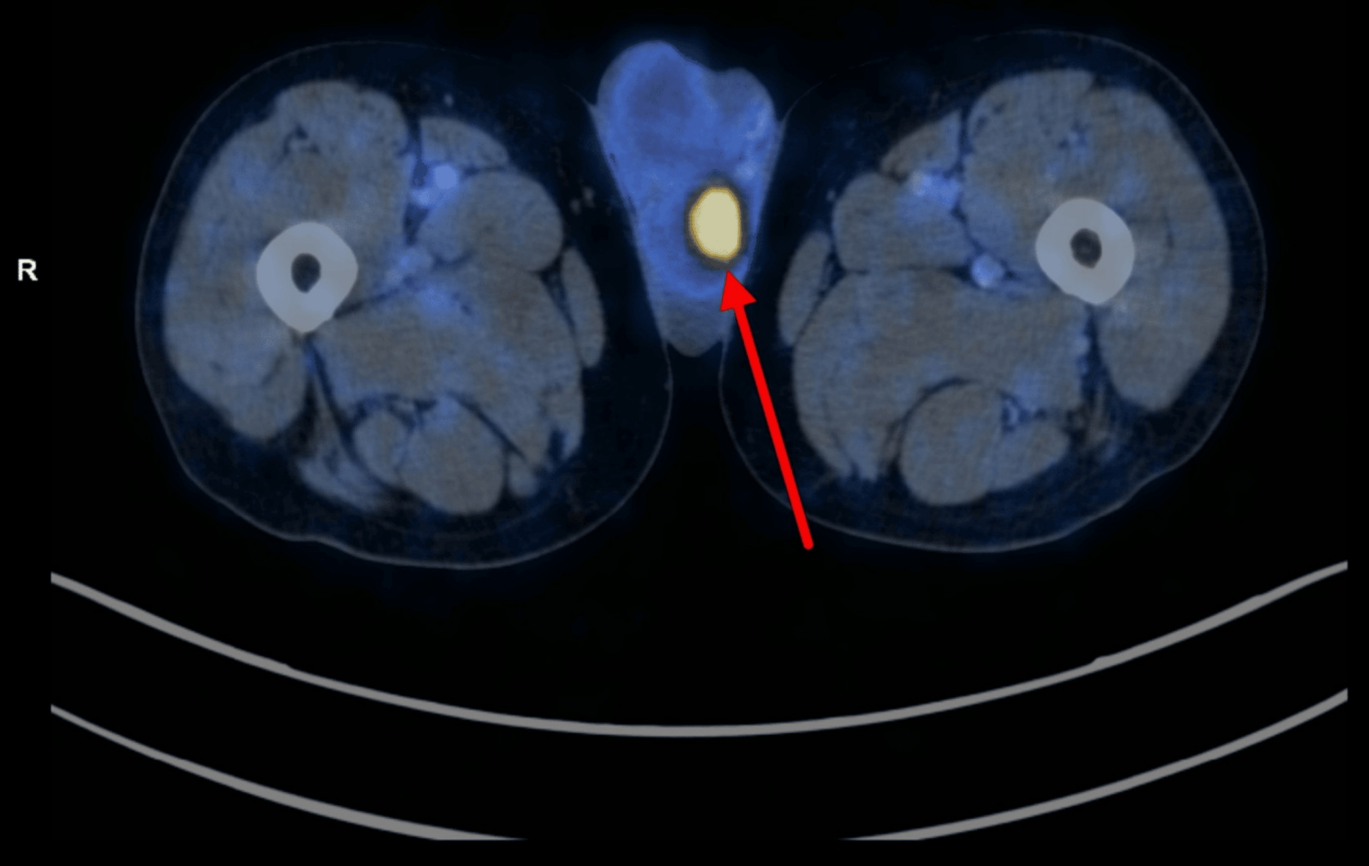
Fused PET-CT axial slice showing a focally intense PSMA-avid lesion in the left testicular region. PET, positron emission tomography; PSMA, prostate-specific membrane antigen; CT, computed tomography

Based on these findings, the patient underwent whole-body gallium-68 prostate-specific membrane antigen positron emission tomography-computed tomography (^68^Ga-PSMA PET-CT), which demonstrated a PSMA-avid, heterogeneously enhancing lesion involving almost the entire prostate gland, with infiltration into the bilateral seminal vesicles and urinary bladder (maximum standardized uptake value {SUVmax}: initial, 19.3; delayed, 24.3). In addition, two enhancing focal lesions were identified in the left testis (SUVmax: 14.0), thereby upstaging the disease to cT4N0M1c (Figures 1, 2).

**Figure 3:**
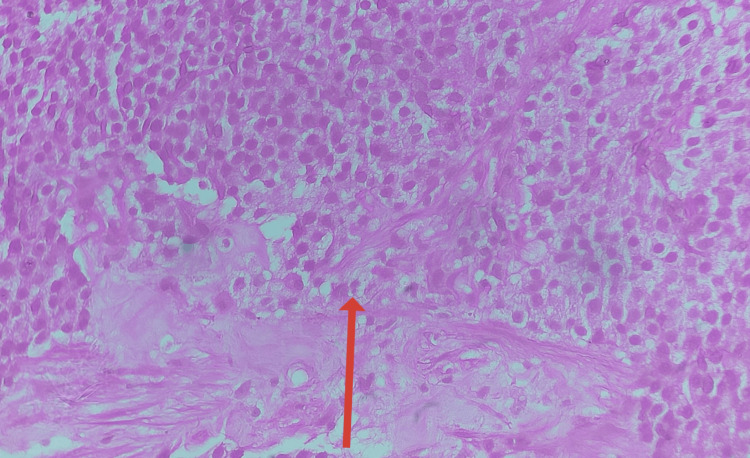
TRUS-guided biopsy of the prostate showing evidence of prostate adenocarcinoma. TRUS: transrectal ultrasound

A transrectal ultrasound (TRUS)-guided prostate biopsy, consisting of 12 systematic cores, confirmed the presence of high-grade prostatic acinar adenocarcinoma. Tumor involvement ranged from 35% to 85% across the sampled cores, with several cores showing complete infiltration. The malignant glands were arranged in sheets, trabeculae, cords, and lobules, with poorly formed glands, focal cribriform architecture, and occasional solid nests. Most involved cores demonstrated a Gleason score of 5 + 5 = 10, with a few showing 5 + 4 = 9, corresponding to Grade Group 5. Perineural invasion was identified in one core, while lymphovascular invasion was not observed. Overall, the biopsy features were consistent with an extensively infiltrative, poorly differentiated adenocarcinoma (Figure 3).

In view of the PET-CT findings, the patient underwent bilateral high orchidectomy in December 2023, with a high inguinal approach on the left side and a low approach on the right.

**Figure 4:**
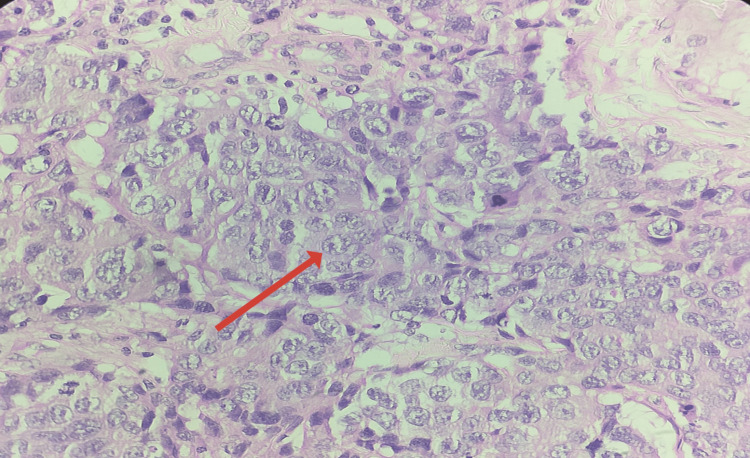
Histopathological examination of testicular biopsy sample showing metastatic deposits of prostate adenocarcinoma.

**Figure 5:**
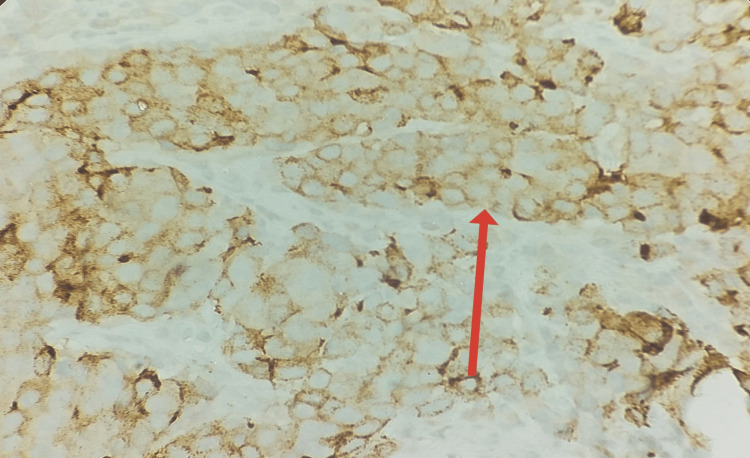
Immunohistochemistry demonstrates strong cytoplasmic granular positivity for AMACR (P504S) in tumor cells (×40).

Histopathological examination and immunohistochemistry confirmed metastatic deposits of prostate adenocarcinoma within the left testis, revealing five metastatic foci, the smallest measuring 1.5 × 1 × 0.5 cm and the largest 1.8 × 1.5 × 0.5 cm, infiltrating carcinoma arranged in sheets, glands with focal cribriforming and nests between seminiferous tubules. Marked cytological atypia with vesicular nuclei, prominent nucleoli, frequent mitoses, and comedo necrosis were noted. The tumor involved the epididymis but remained confined to the testis. The rete testis and spermatic cord margins were free of tumor. Lymphovascular tumor emboli were present (Figures 4, 5). No metastatic involvement was noted on the right side.

Following surgery, the patient was started on abiraterone, along with prednisolone as part of systemic therapy intensification to suppress residual adrenal and intratumoral androgen production. At four months following surgery, his serum PSA had gradually declined to 4.63 ng/mL (reference range: 0.0-4.4).

## Discussion

Testicular metastases from prostate carcinoma represent an exceptionally rare clinical finding. Autopsy studies first demonstrated that secondary tumors of the testis occur infrequently, with early series highlighting their low overall incidence among solid tumors [1]. Subsequent analyses reinforced this rarity, showing that metastatic testicular involvement typically arises in the setting of disseminated malignancy and is often discovered incidentally rather than through symptoms [3]. A comprehensive autopsy series of patients with prostate cancer further confirmed that testicular involvement occurs in only a small fraction of cases, even among those with widespread metastatic disease [2].

Testicular metastases are uncommon because several anatomic and physiologic characteristics make the testes an inhospitable site for secondary tumor implantation. The lower scrotal temperature is thought to impair the survival and proliferation of metastatic cells [1-3]. Additionally, the blood-testis barrier formed by Sertoli cell tight junctions restricts the hematogenous and lymphatic entry of malignant cells, creating an immunologically privileged environment resistant to metastatic seeding [7,8]. Moreover, the venous and lymphatic drainage patterns of the prostate typically direct flow centrally rather than caudally toward the testes. These factors collectively contribute to the very low incidence of testicular metastasis observed in autopsy and clinical series [1-3].

Because most lesions are clinically silent, the symptomatic metastatic involvement of the testis is primarily documented in isolated case reports and limited clinical series [4-6]. These cases frequently involve high-grade or advanced prostate cancer, supporting the notion that testicular metastasis reflects aggressive tumor biology.

The present case illustrates an unusual metastatic pattern in prostate adenocarcinoma, with testicular involvement detected incidentally during staging evaluation. The patient had high-grade (Gleason 10) disease with locally advanced features and was ultimately staged as cT4N0M1c. Functional imaging revealed distinct PSMA-avid lesions within the left testis, prompting surgical confirmation. Histopathology demonstrated multiple metastatic foci infiltrating the testicular parenchyma, reflecting the aggressive biology of the tumor. This pattern of spread, although rare, emphasizes the importance of comprehensive staging in advanced prostate cancer, particularly when high-grade features and biochemical progression are present.

Advancements in molecular imaging have improved the ability to detect unusual metastatic sites. PSMA PET-CT has shown superior performance compared to standard imaging modalities in both staging and restaging prostate cancer, as demonstrated in multicenter randomized trials [9]. Furthermore, PSMA PET-CT has proven valuable in identifying small-volume or anatomically atypical metastases that might be missed on ultrasound or CT, including those within the testis [10]. Consensus guidelines have further established standardized approaches for interpreting PSMA PET-CT findings, enhancing its reliability in advanced-stage disease assessment [11]. While PSMA PET-CT has demonstrated superior sensitivity compared to conventional imaging in various clinical settings, its use as a universal or routine modality for all stages of prostate cancer should be interpreted cautiously, especially in the context of a single case report. In our case, however, the modality appropriately complemented clinical and MRI findings and contributed meaningfully to accurate staging and treatment planning.

Historically, testicular metastasis has been viewed as a marker of disseminated systemic disease and associated with poorer prognosis [11]. Although the metastatic burden in the testis represents only a small fraction of the overall disease volume, the recognition of such atypical spread remains clinically relevant. Early identification may prompt the timely initiation or intensification of systemic therapy. In this case, the patient underwent bilateral orchidectomy, followed by the initiation of androgen-signaling inhibition with abiraterone and prednisolone, resulting in a favorable biochemical response, evidenced by a decline in PSA to 4.63 ng/mL at four-month follow-up.

Overall, this case highlights the importance of maintaining a broad differential when evaluating new or unexpected imaging findings in patients with advanced prostate carcinoma. Although rare, testicular metastasis should be considered, particularly in the setting of high-grade tumors and biochemical progression. Functional imaging modalities such as PSMA PET-CT, when used judiciously, may facilitate the earlier recognition of atypical metastatic patterns and contribute to comprehensive staging and individualized management strategies.

## Conclusions

This case highlights an uncommon metastatic pattern in advanced prostate adenocarcinoma, with testicular involvement detected incidentally through functional imaging. The identification of PSMA-avid testicular lesions in a patient with high-grade, locally advanced disease (cT4N0M1c) underscores the importance of comprehensive staging when evaluating aggressive tumors or biochemical progression. Although testicular metastasis represents only a small component of the overall disease burden, early recognition can guide appropriate therapeutic planning. In this patient, bilateral orchidectomy, followed by androgen-signaling inhibition with abiraterone and prednisolone, led to a favorable biochemical response, demonstrated by a decline in PSA levels at follow-up. Functional imaging modalities such as PSMA PET-CT, when used judiciously, may assist in detecting atypical metastatic sites and support individualized management strategies in advanced prostate cancer.
